# Supplementary material for: Validated respiratory drug deposition predictions from 2D and 3D medical images with statistical shape models and convolutional neural networks
Source: PLoS One. 2024 Jan 26;19(1):e0297437. doi: 10.1371/journal.pone.0297437 (PMC10817191; doi:10.1371/journal.pone.0297437)
Supplement: S1 File — Supplementary methodological details and results. We detail our manual cleanup of the upper airways in the Southampton/Air Liquide dataset. We then compare qualitative differences between a DRR and real chest X-ray to understand potential challenges working with real chest X-ray images. We provide results on the two CNN architectures tested for airway segmentation, including DICE coefficient and inference time. We provide additional results on SSAM reconstruction accuracy (reported as dimensional values). We then provide results that justify the choice of distal airway diameter model for the SSAM-reconstructed airways. (PDF) [file pone.0297437.s001.pdf]

Supplementary material

Josh Williams[1,2], Haavard Ahlqvist[1], Alexander Cunningham[1], Andrew Kirby[3], Ira Katz[4], John Fleming[5,6], Joy Conway[5,7], Steve Cunningham[8], Ali Ozel[1*], and Uwe Wolfram[1,9*]

[1]School of Engineering and Physical Sciences, Heriot-Watt University, Edinburgh, UK
[2]Hartree Centre, STFC Daresbury Laboratory, Daresbury, UK
[3]Royal Hospital for Children and Young People, NHS Lothian, Edinburgh, UK
[4]Consultant, Meudon, France
[5]National Institute of Health Research Biomedical Research Centre in Respiratory Disease, Southampton, UK
[6]Department of Medical Physics and Bioengineering, University Hospital Southampton NHS Foundation Trust, Southampton, UK
[7]Respiratory Sciences, Centre for Health and Life Sciences, Brunel University, London, UK
[8]Centre for Inflammation Research, University of Edinburgh, Edinburgh, UK
[9]Institute for Material Science and Engineering, TU Clausthal, Clausthal-Zellerfeld, Germany

December 30, 2023

* UW and AO share last authorship.

Address correspondence to josh.williams@stfc.ac.uk

1 Manual cleanup of upper airways

To mitigate the issues in image acquisition discussed by Conway et al. (2012), we had to manually clean the throat to allow passage for the air and particles to travel through which would be close to expected *in vivo* conditions during inhalation. We extracted a baseline upper airway segmentation using a semi-automatic region-growing approach implemented in Python. The baseline segmentation was imported into 3D Slicer, where the glottal cross-sectional area was filled in manually with a paintbrush tool to better agree with upper airway morphologies in literature (Feng et al., 2018; Zhao et al., 2020; Scheinherr et al., 2015). The segmentation was then smoothed to remove artificial bumps introduced by the paintbrush tool. Visual representations of two corrected airways are shown in Figure S1.

2 Comparing digitally reconstructed radiographs to real X-ray images

One limitation of our validation study is the use of digitally reconstructed radiographs (DRRs) instead of true chest X-ray images. Here we briefly discuss the differences in images produced with these approaches, to understand how this would affect the generalisation to true X-rays when applied in a real-world clinical setting. In Figure S2, we compare two X-ray images from the same patient (one real X-ray image and one DRR). These images are taken at different time instants, the patient may have been given different instructions or be in a different position, and the images have not been registered which does not allow direct quantitative comparison. One key difference is that the real X-ray image captures much more detail than the DRR. The real X-ray had 2022×2022 pixels, whereas the CT used to create the DRR had $512 \times 512 \times 145$ voxels, meaning the resultant DRR would have capture less details.

The increased level of detail can be seen in the intensity profiles shown in Figure S3. Both profiles follow the same general trend, as the DRR matches the peaks and troths of the real X-ray profile. The real X-ray profile appears much noisier, due to the additional detail seen in Figure S2a, which is the main difference between the two sets of results. Difficulties in fitting the SSAM to noisier data than observed in training may require the use of Robust PCA

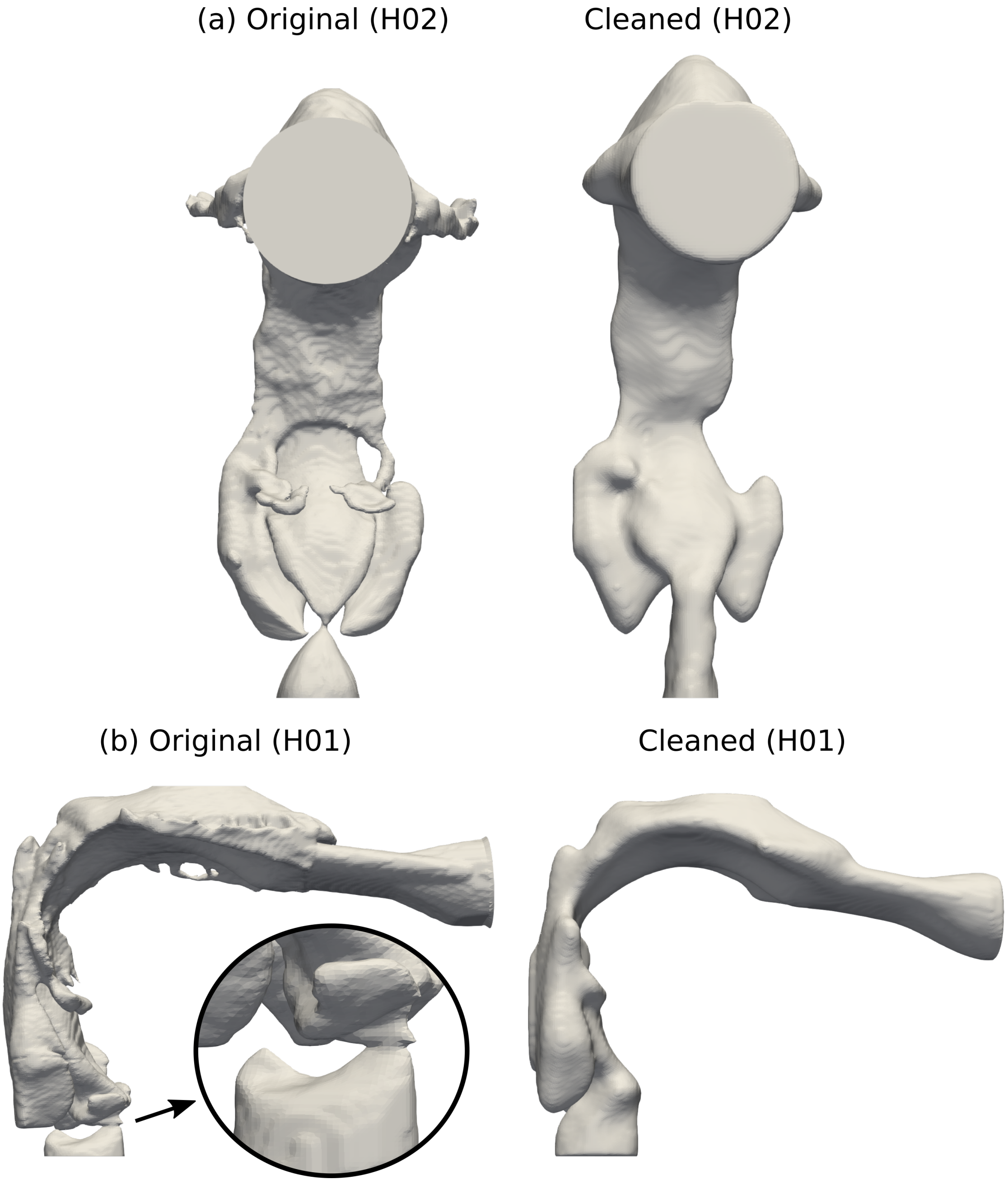

Figure S1: Visualisation of upper airway surface mesh before and after manual cleanup for two cases (Conway et al., 2012). The upper row shows the most constricted case (H02). The lower row shows a less extreme, but still heavily constricted case with a zoomed in view.

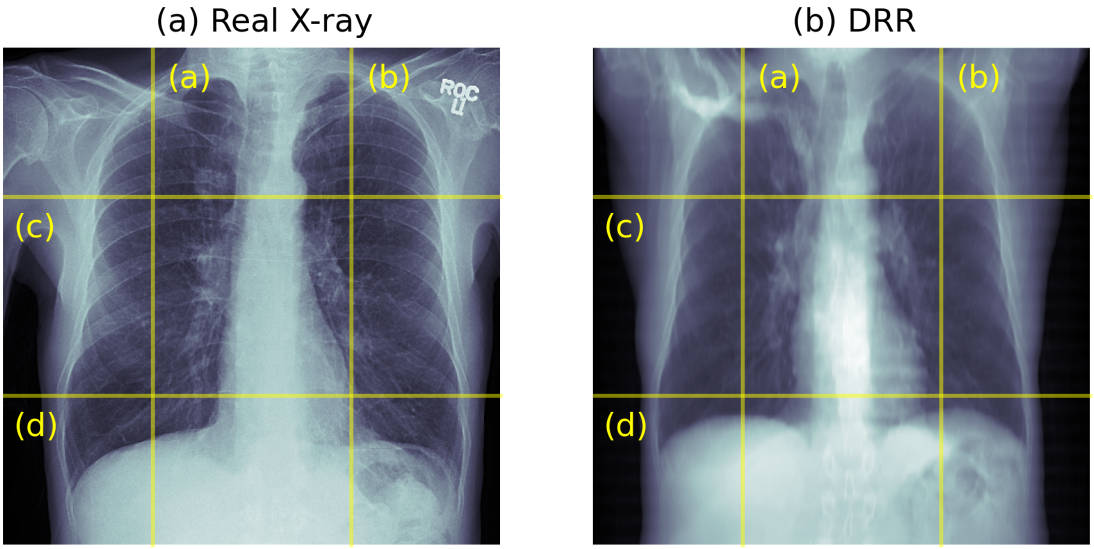

Figure S2: Comparison of (a) real X-ray and (b) a DRR which was reconstructed from CT data of the same patient. Annotations correspond to subplots in Figure S3. Patient data was taken from The Cancer Imaging Archive (Armato III et al., 2011; Clark et al., 2013).

in future studies (De la Torre and Black, 2001). In Figure S3c, the intensity at the boundaries of the profile is significantly different, which appears due to the differences in patient arm placement in Figure S2. However, the SSAM does not model pixels in this region and is therefore unaffected by such differences.

3 CNN architecture analysis

To find the optimal loss function γ parameter in Equation 11, we trained three U-Net CNNs with $\gamma = \{1, 3, 5\}$ (Figure S4). By comparing the DICE coefficient over the 10 cases in the validation set, we observed $\gamma = 1$ to produce the lowest minimum DICE coefficient (0.85). When $\gamma = 3$ and $\gamma = 5$, the minimum and maximum DICE coefficients were approximately the same (0.855 minumum and 0.95 maximum). Throughout the remainder of the study, we chose $\gamma = 5$ in our loss function due to the slightly higher lower quartile, median and upper quartile (all approximately 1% larger for $\gamma = 5$).

As the U-Net has a large memory consumption, we also trained an ENet CNN architecture as a less computationally expensive alternative (Figure S5a). Both architectures were trained using $\gamma = 5$ and using 25 augmentations (with rotation $\pm 15°$). There was a significant difference in DICE coefficient ($p < 0.05$), as the U-Net median and upper-quartile was

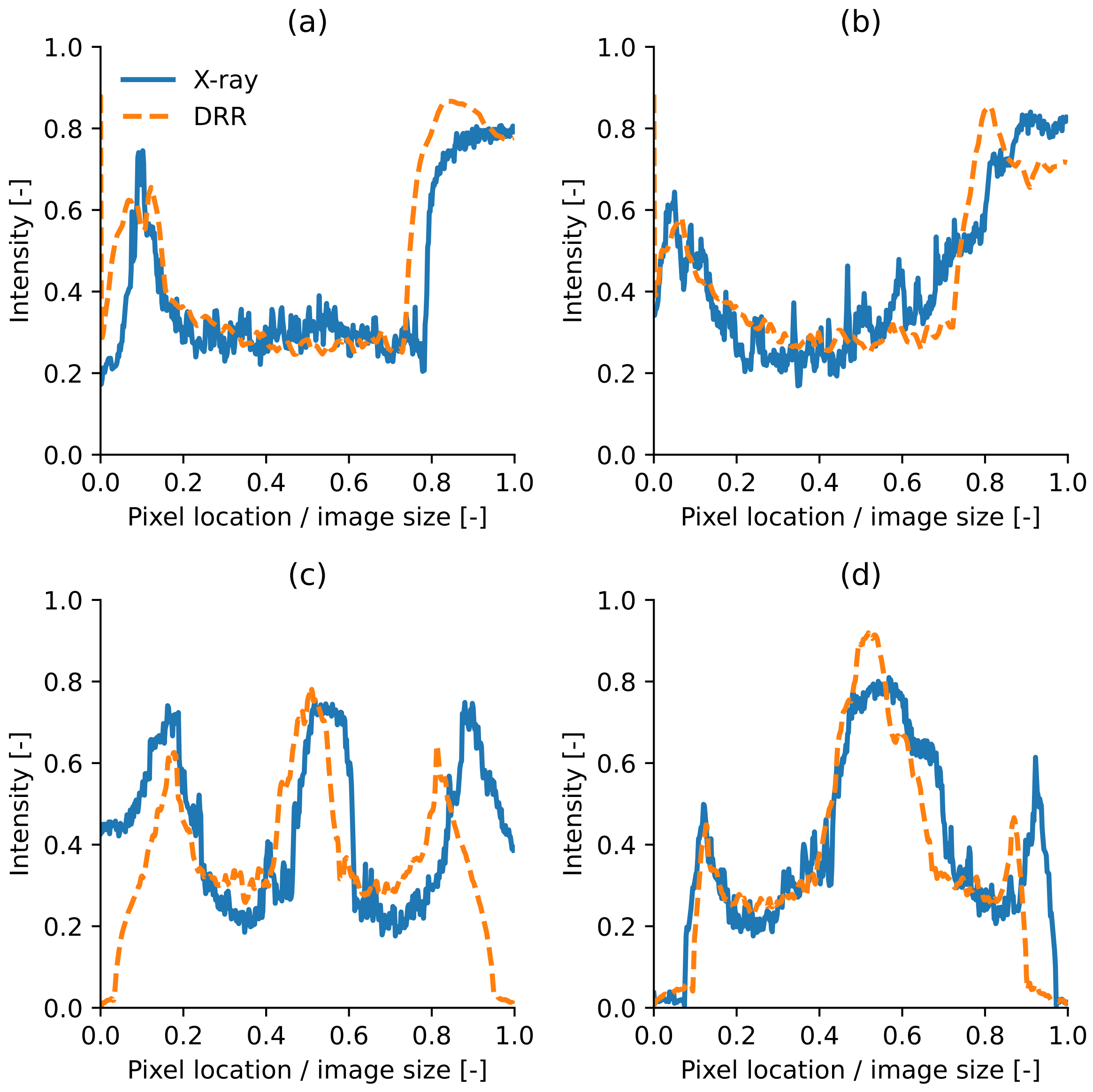

Figure S3: Pixel intensity profile at four lines shown in Figure S2.

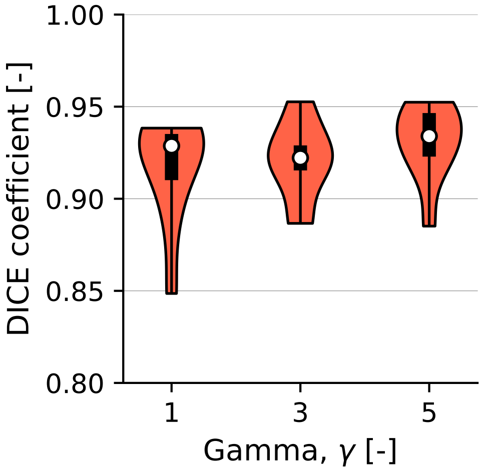

Figure S4: Sensitivity analysis of focal loss parameter γ in Equation 11 to optimise CNN DICE coefficient. CNNs were trained with no augmentations and results were computed from 10 scans in validation set.

4% larger than the ENet. The U-Net lower-quartile was 4.7% larger than the E-Net. We also compared the time-taken for one forward-pass through the networks (inference time), for all images in the training and validation dataset (Figure S5b). For the smallest images in the dataset (number of voxels below the 25th percentile), the mean inference time was 5.89 s and 7.81 s for the ENet and U-Net, respectively (1.3 times speed-up with ENet). In contrast, for the largest images in the dataset (number of voxels larger than the 75th percentile), the mean inference time was 15.9 s and 91.28 s for the ENet and U-Net, respectively, which is a speed-up of 5.7 times when using the ENet. As speed is not a significant driver of our developments at the present stage, we use the U-Net as our default segmentation tool for this study due to its improved DICE coefficient. However, due to memory overheads required for inference with the U-Net, we used the ENet to segment airways from the high resolution CT of the Southampton/Air Liquide dataset. Additionally, the ENet DICE coefficient is reasonable and may be preferable for implementation in a clinical workflow, where speed is a key factor.

4 SSAM morphology assessment

Here we provide the results presented in Figures 4 and 6 as scatter plots to allow for comparison of absolute values of lung space volume and airway diameter. We found it important

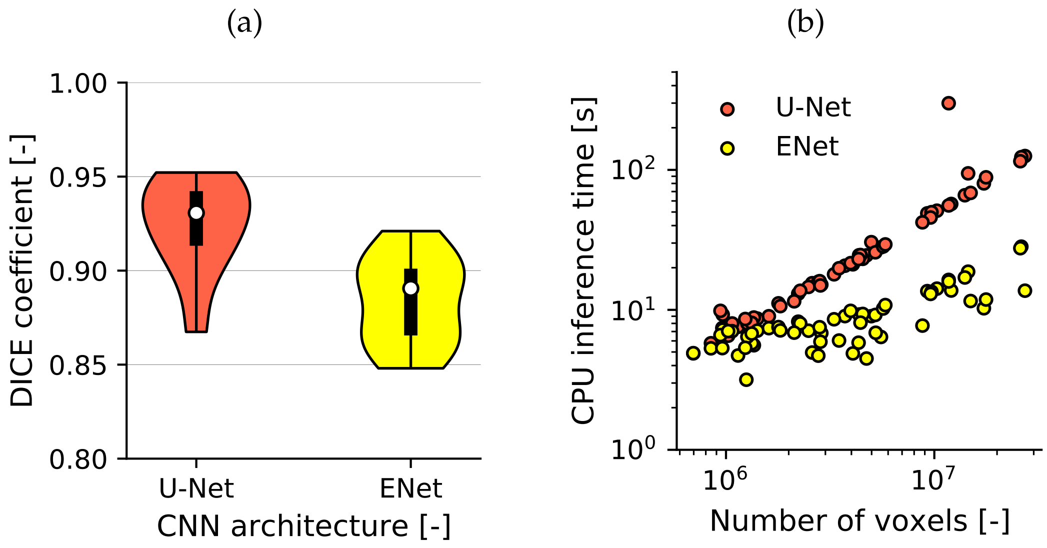

Figure S5: Comparison of accuracy and speed for U-Net and ENet architectures. Panels show (a) DICE coefficient from 10 scans in validation set, and (b) inference time relative to size of image, computed on combined training and validation set.

to provide both plots, to show that the absolute values are within a realistic range. This also shows a clear linear correlation between the ground truth measurements and those predicted by the SSAM. The lung space volume predicted by the SSAM agreed particularly well (concordance correlation coefficient $CCC \approx 0.89$, Figure S6). The diameter predicted by the SSAM (Figure S7) showed a less ideal fit than the lung volume, as $CCC = 0.657$ and 0.71. The fit was slightly improved with two projections, compared to one projection (5.3% increase).

5 Choice of generated airway diameter model

As the SSAM-reconstructed airways do not have the same level of information as the CT-based airways, best practices for generating distal airways may not be the same as that of airways segmented directly from patient CT data. Specifically, on the chest X-ray, only the trachea and main bronchi are visible. Even for these 'visible' airways, the contrast is poor and the quality of reconstructed diameters was found to be varied in our analysis. When using the diameter of the parent branch to assign diameters of the entire airway tree (Bordas et al., 2015), it makes sense that erroneous diameters cause some level of error that propagates into the distal airways. Therefore, we compared various models for computing diameter obtained in literature (Bordas et al., 2015; Montesantos et al., 2016).

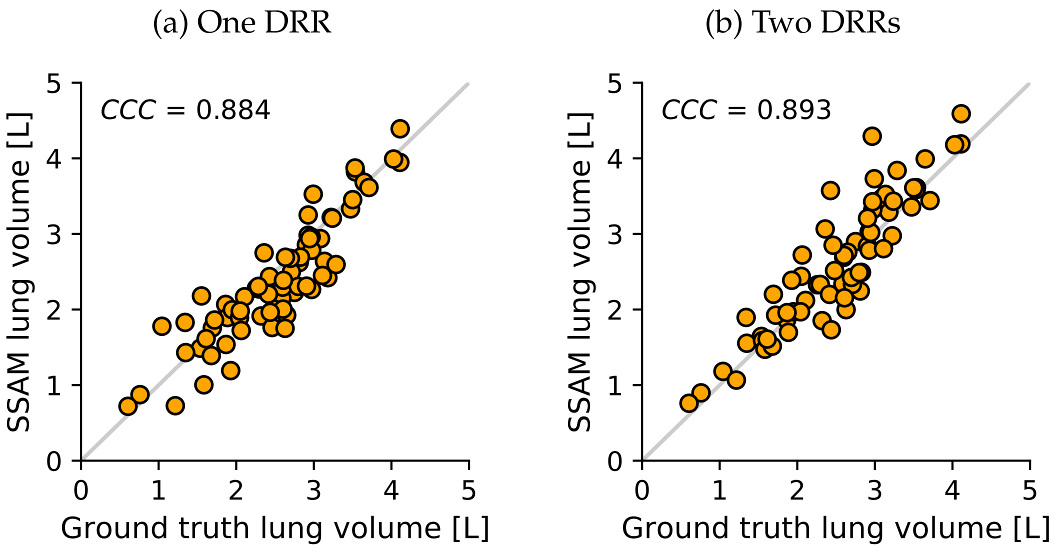

Figure S6: Comparison of lung volume predicted by our SSAM compared to the ground truth segmentations. We show the influence of including an additional projection on lung volume error. Panels show SSAM results with (a) one DRR provided for fitting (anterior-posterior projection), and (b) two DRRs provided (anterior-posterior and lateral projections). *CCC* is the concordance correlation coefficient.

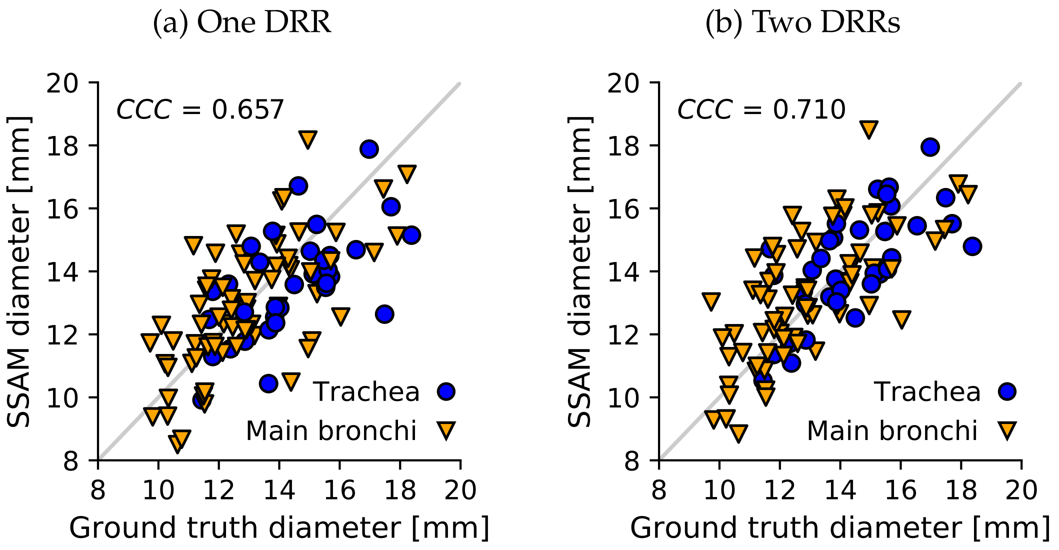

Figure S7: Comparison of airway diameter predicted by our SSAM compared to the ground truth segmentations. We show the influence of including an additional projection on diameter error in the trachea and main bronchi. Panels show SSAM results with (a) one DRR provided for fitting (anterior-posterior projection), and (b) two DRRs provided (anterior-posterior and lateral projections). *CCC* is the concordance correlation coefficient.

Bordas et al. (2015) assigned a logarithmic decay of diameter based on Horsfield order (Horsfield et al., 1976) defined as

$$\log D_i(H_i) = (H_i - N_H)\log(R_d H) + \log(D_N) \quad (1)$$

where D_i is the diameter of branch i, H is the Horsfield order, N_H is the Horsfield order of the reference airway in the image-based domain, D_N is the diameter of the reference airway in the image-based domain, $R_d H = 1.15$ is a constant representing the logarithmic decrease of diameter with Horsfield order (Horsfield et al., 1976; Tawhai et al., 2004; Bordas et al., 2015). This approach requires declaration of an airway segment in the image-based airways to obtain reference values that are used to assign diameter and Horsfield order to the generated airways. Bordas et al. (2015) used the parent branch of the image-based airway that is connected to the generated airway, i (referred to here as 'parent' model). Alternatively, Montesantos et al. (2016) calculated airway diameter based on the airway length as $D_i = L_i/3$, with a cutoff $D_{child} \leq 0.95 D_{parent}$ (referred to here as 'length' model). Ventilation simulations using the 'parent' model was shown to have good agreement with clinical ventilation data in healthy and diseased patients (Bordas et al., 2015). Therefore, we used the parent model for CT-based airways. We used ground truth segmentations with the parent model as a benchmark to evaluate the accuracy of the parent model or length model in on generated airway diameter in SSAM-reconstructed airways.

We found the length model to produce better agreement with the ground truth dataset than the parent model proposed by Bordas et al. (2015) (Figure S8a). This is likely due to difficulty inferring the diameter of the central airways from a chest X-ray image. As can be seen in Figure S8b, the generated airway length showed excellent agreement in the SSAM and ground truth datasets. Based on this, we chose to use the length model of Montesantos et al. (2016) to assign diameters to the full SSAM airway tree.

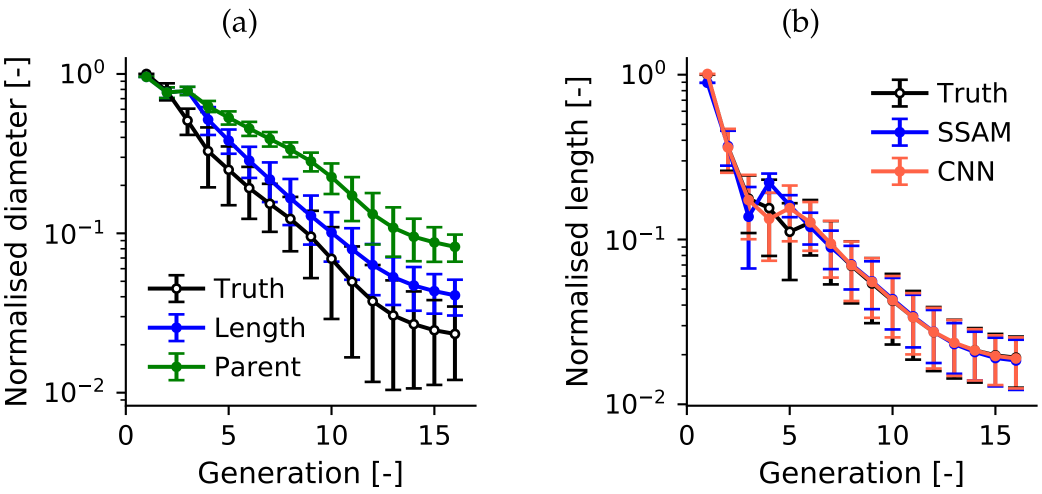

Figure S8: Comparison of diameter error in full conducting airway tree from a SSAM with varying models to calculate diameter. The 'truth' results were generated from ground truth segmentations with the 'parent' model for diameter (Bordas et al., 2015).

References

S. G. Armato III, G. McLennan, L. Bidaut, M. F. McNitt-Gray, C. R. Meyer, A. P. Reeves, B. Zhao, D. R. Aberle, C. I. Henschke, E. A. Hoffman, et al. The lung image database consortium (lidc) and image database resource initiative (idri): a completed reference database of lung nodules on ct scans. *Medical physics*, 38(2):915–931, 2011.

R. Bordas, C. Lefevre, B. Veeckmans, J. Pitt-Francis, C. Fetita, C. E. Brightling, D. Kay, S. Siddiqui, and K. S. Burrowes. Development and analysis of patient-based complete conducting airways models. *PloS one*, 10(12):e0144105, 2015.

K. Clark, B. Vendt, K. Smith, J. Freymann, J. Kirby, P. Koppel, S. Moore, S. Phillips, D. Maffitt, M. Pringle, et al. The Cancer Imaging Archive (TCIA): maintaining and operating a public information repository. *Journal of digital imaging*, 26(6):1045–1057, 2013.

J. Conway, J. Fleming, C. Majoral, I. Katz, D. Perchet, C. Peebles, L. Tossici-Bolt, L. Collier, G. Caillibotte, M. Pichelin, et al. Controlled, parametric, individualized, 2-D and 3-D imaging measurements of aerosol deposition in the respiratory tract of healthy human subjects for model validation. *Journal of aerosol science*, 52:1–17, 2012.

F. De la Torre and M. J. Black. Robust principal component analysis for computer vision. In

Proceedings Eighth IEEE International Conference on Computer Vision. ICCV 2001, volume 1, pages 362–369. IEEE, 2001.

Y. Feng, J. Zhao, C. Kleinstreuer, Q. Wang, J. Wang, D. H. Wu, and J. Lin. An in silico inter-subject variability study of extra-thoracic morphology effects on inhaled particle transport and deposition. *Journal of Aerosol Science*, 123:185–207, 2018.

K. Horsfield, F. G. Relea, and G. Gumming. Diameter, length and branching ratios in the bronchial tree. *Respiration physiology*, 26(3):351–356, 1976.

S. Montesantos, I. Katz, M. Pichelin, and G. Caillibotte. The creation and statistical evaluation of a deterministic model of the human bronchial tree from HRCT images. *PLOS one*, 11(12):e0168026, 2016.

A. Scheinherr, L. Bailly, O. Boiron, A. Lagier, T. Legou, M. Pichelin, G. Caillibotte, and A. Giovanni. Realistic glottal motion and airflow rate during human breathing. *Medical engineering & physics*, 37(9):829–839, 2015.

M. H. Tawhai, P. Hunter, J. Tschirren, J. Reinhardt, G. McLennan, and E. A. Hoffman. Ct-based geometry analysis and finite element models of the human and ovine bronchial tree. *Journal of applied physiology*, 97(6):2310–2321, 2004.

J. Zhao, Y. Feng, and C. A. Fromen. Glottis motion effects on the particle transport and deposition in a subject-specific mouth-to-trachea model: A cfpd study. *Computers in Biology and Medicine*, 116:103532, 2020.
